# Vitiligo et phénomène de Koebner suite à des injections de plasma riche en plaquettes

**DOI:** 10.11604/pamj.2019.32.58.16779

**Published:** 2019-02-04

**Authors:** Mouna Ejjiyar, Mehdi Sahibi, Mehdi El Gueouatri, Abdelkoddous Bhihi, Mehdi Mahrouch, Imane Yafi, Moulay Driss El Amrani, Yassine Benchamkha

**Affiliations:** 1 Service de Chirurgie Plastique, CHU Mohammed VI, Marrakech, Maroc

**Keywords:** PRP, vitiligo, phénomène de Koebner, PRP, vitiligo, Koebner phenomenon

## Abstract

Le vitiligo est un trouble commun et acquis de la pigmentation cutanée caractérisé par une perte sélective et souvent continue de mélanocytes épidermiques. Nous rapportons le cas d'une patiente ayant développé un vitiligo de la face suite à des injections de plasma riche en plaquettes. Le diagnostic de phénomène de Koebner a été retenu pour expliquer la survenue de vitiligo sur les zones mêmes d'injection de plasma riche en plaquettes.

## Abstract (en)

Vitiligo is a common and acquired skin pigmentation disorder characterized by a selective and often continuous loss of epidermal melanocytes. We report the case of a female patient who developed facial vitiligo following platelet-rich plasma injections. The diagnosis of Koebner phenomenon was retained in order to explain the occurrence of vitiligo on the areas where platelet-rich plasma (PRP) had been injected.

## Introduction

Le vitiligo est une dermatose auto-immune caractérisée par l'apparition de macules hypochromiques sur le visage et le corps, intéressant de façon préférentielle les zones de frottement ou sujettes à des micro-traumatismes (phénomène de Koebner). Malgré les différents moyens disponibles, sa prise en charge relève souvent du défi thérapeutique. Récemment, certaines études ont abordé le plasma riche en plaquettes (PRP) comme étant une alternative thérapeutique. Nous rapportons le cas d'une patiente ayant développé un vitiligo suite à des injections de PRP, le facteur déclenchant étant le caractère traumatisant des injections.

## Patient et observation

**Figure 1:**
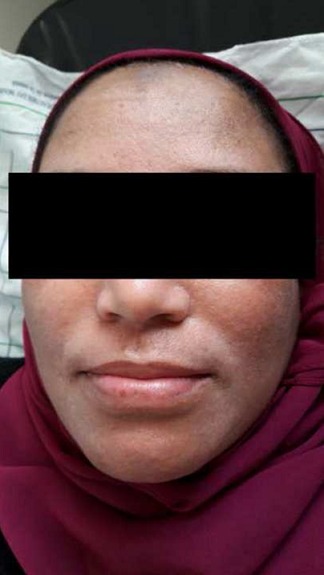
Tâches hyperpigmentées de la face: aspect avant injections de PRP

**Figure 2:**
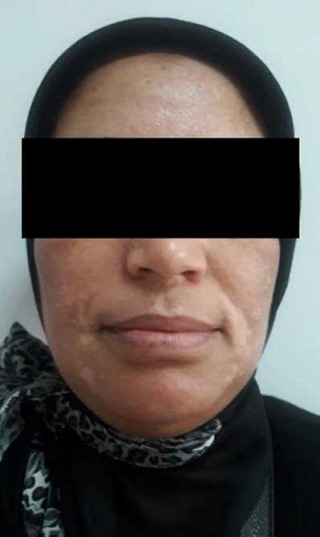
Macules hypochromiques en regard des sillons nasogéniens en rapport avec un vitiligo: aspect après la troisième séance de PRP

**Figure 3:**
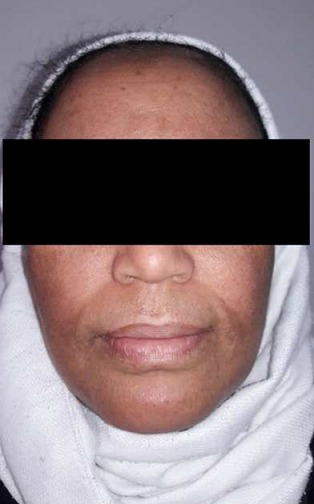
Disparition des macules de vitiligo: aspect après 13 séances de photothérapie

Nous rapportons le cas d'une patiente âgée de 38 ans, ayant un antécédent de brûlure thermique survenue au jeune âge, et qui a gardé comme séquelle des tâches hyperpigmentées au niveau de la face. A l'examen, on retrouvait une patiente de phototype IV à V, avec quelques tâches hyperpigmentées éparpillées au niveau de la face notamment en regard du front et des sillons nasogéniens (Figure 1). L'indication d'injections de PRP en mésothérapie a été posée dans le but d'améliorer la trophicité cutanée et de redonner un coup d'éclat à la peau. Des séances d'injection de PRP espacées d'un mois ont été programmées, avec à chaque séance un prélèvement de 8 cc de sang de la patiente, qui a été centrifugé à 3400 tours pendant 5 minutes, puis injecté en mésothérapie à l'aide d'une seringue fine (25 gauge), en insistant sur les zones hyperpigmentées. Après la troisième séance, la patiente est revue avec un début de dyschromie prédominant en regard des sillons nasogéniens de façon bilatérale. Une consultation en dermatologie a conclu à un vitiligo non segmentaire débutant (Figure 2). Devant cette évolution, les injections de PRP ont été interrompues et la patiente a démarré un traitement par photothérapie. Après 13 séances réalisées à un rythme de deux séances par semaine, nous avons pu constater une disparition totale des tâches dyschromiques (Figure 3). Néanmoins, un traitement complémentaire par 17 séances supplémentaires reste à prévoir pour assurer un meilleur résultat et éviter une éventuelle récidive.

## Discussion

Le vitiligo est un trouble commun et acquis de la pigmentation cutanée pouvant avoir un impact important sur la qualité de vie. Il est caractérisé par une perte sélective et souvent continue de mélanocytes épidermiques. Affectant principalement la peau, les muqueuses et les poils peuvent occasionnellement être atteints. Son incidence reste faible, moins de 1% de la population mondiale est affectée. Ces dernières années, des progrès considérables ont été réalisés pour comprendre le rôle de la génétique dans le vitiligo avec plusieurs gènes de susceptibilité identifiés. L'hypothèse auto-immune de la pathogenèse du vitiligo n'est également pas à écarter [1]. Le vitiligo non segmentaire, forme commune et celle retrouvée chez notre patiente, est caractérisé par des macules hypochromiques bilatérales et souvent symétriques. Les lésions ont une distribution acrofaciale (mains et pieds, atteinte péri-orificielle faciale) ou sont dispersées symétriquement sur tout le corps et évoluent de manière imprévisible. Chez notre patiente, les macules étaient localisées en regard des sillons nasogéniens de façon bilatérale. La topographie peut correspondre aux zones de frottement ou de pression continue sur la peau, connue sous le nom de phénomène de Koebner [2]: il est défini par la répartition préférentielle de la dépigmentation sur les zones subissant fréquemment des micro-traumatismes. Ceci peut donc expliquer l'apparition soudaine du vitiligo chez notre patiente, et plus précisément en regard des sillons nasogéniens, zones d'injection du PRP. Le diagnostic de vitiligo est clinique, et son traitement repose, outre les techniques de camouflage, sur les anti-inflammatoires topiques, la photothérapie comme chez notre patiente, les topiques combinés aux UV, le laser CO2, les corticostéroïdes oraux, les statines [3] et rarement les autres agents immunosuppresseurs [2]. La phytothérapie constitue la médecine alternative de référence. Dans son enquête, Ait Ouakrouch [4] a révélé pas moins de 25 plantes recensées et présumées posséder des propriétés contre le vitiligo. Néanmoins, leur utilisation conventionnelle doit être rationnalisée en raison de leur richesse en composants actifs. L'utilisation du PRP seul ou en préparation sous forme de gel associant de l'acide hyaluronique [5], voire même en intraveineux a été décrite dans quelques études, ayant démontré son efficacité en matière de repigmentation dans le vitiligo. En définition, le PRP est une préparation autologue de plaquettes dans du plasma concentré. C'est le procédé de médecine régénératrice le plus simple et le moins cher utilisé avec succès dans plusieurs domaines et notamment en dermatologie, en raison de sa facilité d'utilisation et de sa biosécurité, ainsi que sa forte concentration en facteurs de croissance et en cellules souches.

## Conclusion

Ainsi, nous pouvons conclure que l'apparition de façon brusque d'un vitiligo non segmentaire chez notre patiente était due aux micro-traumatismes répétitifs induits par les injections de PRP au niveau des zones mêmes où les macules hypochromiques ont été découvertes, rentrant ainsi dans le cadre du phénomène de Koebner.

## Conflits d’intérêts

Les auteurs ne déclarent aucun conflit d'intérêts.
